# CT-Based Habitat Radiomics Combining Multi-Instance Learning for Early Prediction of Post-Neoadjuvant Lymph Node Metastasis in Esophageal Squamous Cell Carcinoma

**DOI:** 10.3390/bioengineering12080813

**Published:** 2025-07-28

**Authors:** Qinghe Peng, Shumin Zhou, Runzhe Chen, Jinghui Pan, Xin Yang, Jinlong Du, Hongdong Liu, Hao Jiang, Xiaoyan Huang, Haojiang Li, Li Chen

**Affiliations:** 1 Guangdong Esophageal Cancer Institute, Sun Yat-sen University Cancer Center, Guangzhou 510060, China; pengqh@sysucc.org.cn (Q.P.); yangx@sysucc.org.cn (X.Y.); liuhd@sysucc.org.cn (H.L.); 2 Department of Radiation Oncology, State Key Laboratory of Oncology in South China, Collaborative Innovation Center for Cancer Medicine, Sun Yat-sen University Cancer Center, Guangzhou 510060, China; runzhe.chen@outlook.com (R.C.); dujl@sysucc.org.cn (J.D.); huangxy@sysucc.org.cn (X.H.); 3 Department of Radiology, State Key Laboratory of Oncology in South China, Collaborative Innovation Center for Cancer Medicine, Sun Yat-sen University Cancer Center, Guangzhou 510060, China; zhousm@sysucc.org.cn; 4 Department of Radiation Oncology, Renmin Hospital, Wuhan University, Wuhan 430060, China; panjinghui@whu.edu.cn; 5 School of Electronic Information, Wuhan University, Wuhan 430064, China; jh@whu.edu.cn

**Keywords:** esophageal squamous cell carcinoma (ESCC), computed tomography (CT), radiomic nomogram, lymph node metastasis (LNM), neoadjuvant therapy (NAT)

## Abstract

Early prediction of lymph node metastasis (LNM) following neoadjuvant therapy (NAT) is crucial for timely treatment optimization in esophageal squamous cell carcinoma (ESCC). This study developed and validated a computed tomography-based radiomic model for predicting pathologically confirmed LNM status at the time of surgery in ESCC patients after NAT. A total of 469 ESCC patients from Sun Yat-sen University Cancer Center were retrospectively enrolled and randomized into a training cohort (*n* = 328) and a test cohort (*n* = 141). Three signatures were constructed: the tumor-habitat-based signature (Habitat_Rad), derived from radiomic features of three tumor subregions identified via K-means clustering; the multiple instance learning-based signature (MIL_Rad), combining features from 2.5D deep learning models; and the clinicoradiological signature (Clinic), developed through multivariate logistic regression. A combined radiomic nomogram integrating these signatures outperformed the individual models, achieving areas under the curve (AUCs) of 0.929 (95% CI, 0.901–0.957) and 0.852 (95% CI, 0.778–0.925) in the training and test cohorts, respectively. The decision curve analysis confirmed a high net clinical benefit, highlighting the nomogram’s potential for accurate LNM prediction after NAT and guiding individualized therapy.

## 1. Introduction

Esophageal carcinoma (EC) is the eighth most common cancer globally and the sixth leading cause of cancer-related death [1]. Esophageal squamous cell carcinoma (ESCC) is the predominant subtype, making up over 90% of EC cases in high-risk regions like China [2,3]. Neoadjuvant therapy (NAT), followed by surgery, is the standard treatment for resectable locally advanced ESCC [4,5]. Lymph node metastasis (LNM) after neoadjuvant chemotherapy (NACT) or chemoradiotherapy (NACRT) significantly influences ESCC prognosis and guides personalized perioperative therapy [6,7,8]. Thus, accurately identifying LN involvement post-NAC is critical.

In clinical practice, computed tomography (CT) is the standard method for diagnosing LNM in ESCC patients receiving NAT [9]. A common diagnostic criterion is a lymph node short-axis diameter over 10 mm, yet only 8.0–37.5% of metastatic nodes in esophageal carcinoma meet this threshold [10]. CT detects metastatic nodes at a rate of 57.14%, significantly lower than the pathological rate of 87.6%, resulting in less-than-ideal accuracy, specificity, and sensitivity [11]. Although factors like the tumor size, depth of tumor invasion, histological type, and neutrophil–lymphocyte ratio have been linked to LNM [12], their reliability remains controversial.

Radiomics can quantitatively describe tissue heterogeneity, objectively capturing characteristics not visually discernible by extracting quantitative features from medical images with high throughput [13]. Recent studies have demonstrated the potential of radiomics and deep learning for predicting LNM in esophageal cancer. A systematic review by Ma et al. integrated the data from nine studies involving 719 patients and found that radiomic models utilizing CT, PET, and MRI achieved a sensitivity of 72% and a specificity of 76% (AUC = 0.74) for predicting LNM in ESCC patients [14]. Studies show that combining radiomic features with clinical risk factors enhances the accuracy of predicting LNM in esophageal cancer compared to using either alone [15].

However, prior research has primarily concentrated on patients eligible for direct surgical intervention, and there has been no exploration of radiomic analysis for predicting LNM status at the time of surgery in ESCC patients who have undergone neoadjuvant therapy. Additionally, traditional radiomic analysis generally considers the tumor as a single entity, often neglecting the phenotypic differences that exist within its subregions [16]. The habitat approach—which segments tumors into distinct subregions by clustering voxels with similar imaging features—has demonstrated potential for more effectively capturing and characterizing intratumoral heterogeneity [17,18]. Furthermore, 2.5D deep learning methods, which leverage adjacent slices to extract localized 3D information at lower computational cost than full 3D approaches, have shown promising results in medical image classification [19,20] but have not been applied to predict LNM in esophageal cancer.

This study aims to develop and validate a CT-based predictive model for pathologically confirmed LNM status at the time of surgery after NAT in patients with locally advanced ESCC, facilitating personalized treatment decisions and prognostic assessment. To ensure reliability and applicability, we employ a focused approach comparing three key modeling strategies: habitat-based radiomic analysis, 2.5D deep learning with multi-instance learning integration, and combined models incorporating clinicoradiological factors.

## 2. Materials and Methods

### 2.1. Patients and Study Design

The retrospective study was ethically reviewed and received approval from the Institutional Review Board (IRB) of Sun Yat-sen University Cancer Center (No. B2021-335-01), and the requirement for informed consent was waived. A total of 469 patients with advanced ESCC who underwent NAT between March 2010 and June 2021 were identified from the institutional database. The inclusion criteria were as follows: (1) patients who underwent NACT or NACRT followed by radical resection with lymph node (LN) dissection; (2) contrast-enhanced CT examinations prior to NAT; (3) histologically confirmed ESCC; (4) detailed pathology records of LNs. The exclusion criteria were as follows: (1) distant metastasis at initial diagnosis; (2) presence of other types of primary tumors; (3) patients who did not undergo surgery within six months of the completion of NAT; (4) incomplete clinical and imaging data records and lack of histological confirmation.

All the enrolled patients were randomly divided into a training cohort (*n* = 328) and a test cohort (*n* = 141) at a ratio of 7:3. To ensure robust model training and hyperparameter optimization, we employed 5-fold cross-validation within the training cohort for all the modeling approaches. The Grid-Search algorithm was utilized to identify the optimal hyperparameters and optimize the algorithms. The final model performance was evaluated on the independent test set. All the enrolled patients underwent radical esophagectomy within six months of the completion of NAT. The clinical endpoint of this study was the pathologically confirmed presence of lymph node metastasis (LNM) at the time of surgery. The patients were categorized into LNM (LN+) or non-LNM (LN−) groups based on the postoperative pathology results.

**Figure 1:**
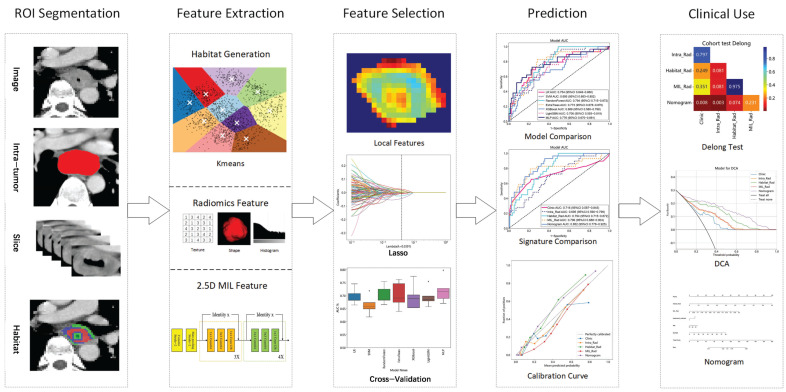
Workflow of this study. Tumor segmentation and regions of interest (ROIs) delineation were conducted by two experienced radiologists. Handcrafted radiomic features were extracted from tumor subregions formed via the Kmeans clustering method to construct a habitat-based radiomic signature (Habitat_Rad). The 2.5−dimension (2.5D) data consisted of the largest cross-section of the tumor and four adjacent CT slices, which were input into three deep learning (DL) architectures, resulting in 15 2.5D DL models. The multiple instance learning radiomic signature (MIL_Rad) was established by integrating features from these DL models, using the predict likelihood histogram (PLH) and bag of words (BoW) methods. A radiomic nomogram was developed by combining the Habitat_Rad and MIL_Rad signatures with independent clinico−radiological factors. The performance of the radiomic nomogram was evaluated using the area under receiver operating characteristic curve (AUC), DeLong test, calibration curve, and decision curve analysis (DCA).

**Figure A1:**
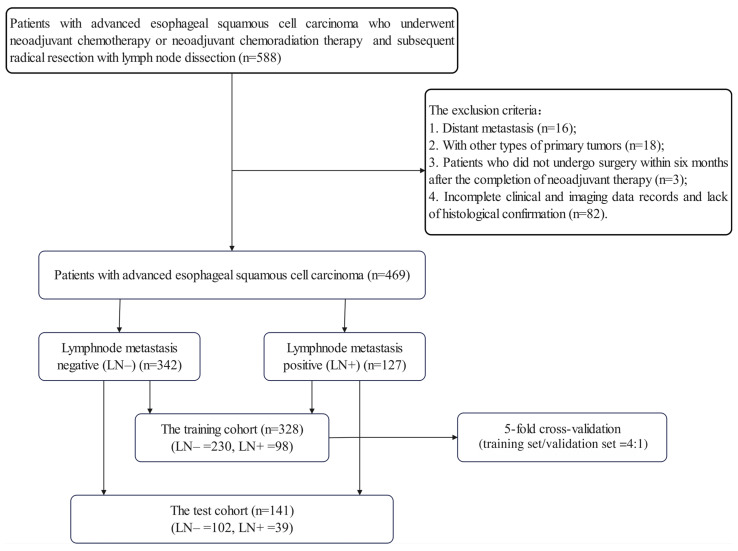
Patient recruitment flow chart. LN+, positive lymph node metastasis; and LN−, negative lymph node metastasis.

Details of patient enrollment are shown in Figure A1, and the overall workflow of this study is illustrated in Figure 1.

### 2.2. Neoadjuvant Regimens and Clinicoradiological Data

All the subjects in this study received a standardized and comprehensive neoadjuvant treatment (NAT) regimen, which included either neoadjuvant chemoradiotherapy or neoadjuvant chemotherapy alone, consistent with the National Comprehensive Cancer Network (NCCN) guidelines from 2010 to 2021 (Appendix A.1). Each patient underwent radical esophagectomy within six months of completing the NAT; the specific surgical procedures are outlined in Appendix A.2.

Clinical factors, including gender, age, smoking and drinking history, treatment method for NAT, clinical T (cT) and clinical N (cN) stages based on the 8th edition of the American Joint Committee on Cancer TNM staging system [21], and key CT features based on the radiologists’ diagnosis (e.g., primary tumor maximum diameter, enhancement pattern, and lymph node characteristics such as maximum short-axis diameter, fusion, extracapsular invasion, and necrosis) were extracted from medical records. These clinical factors and CT features are collectively termed clinicoradiological features. All the patients were categorized by postoperative pathology into LNM (LN+) or non-LNM (LN−) groups.

### 2.3. Image Acquisition and Preprocessing and Tumor Segmentation

All the patients underwent contrast-enhanced CT examination within 2 weeks prior to neoadjuvant therapy using our hospital’s CT scanning systems (Discovery CT750 HD, GE Healthcare; Aquilion TSX-101A, Toshiba; SOMATOM Force; Brilliance iCT, Philips; uCT780, United Imaging Healthcare). The scanning coverage ranged from the thoracic inlet to the costophrenic angle, with the lower edge positioned at 2–7 cm. Following a routine non-enhanced CT scan, contrast-enhanced CT scanning commenced 25 s after administering 1.0 mL/kg of a non-ionic iodine contrast agent intravenously at a rate of 3.0 mL/s via a high-pressure auto-injector. The CT parameters were as follows: a peak voltage of 120 kVp, a tube current of 100–300 mA, a field of view (FOV) of 400–500 mm, a slice thickness of 5 mm, slice spacing of 5 mm, and a matrix of 512 × 512 mm. The raw data were reconstructed at a slice thickness of either 1.0 or 1.25 mm.

Two experienced radiologists with over 10 years of esophageal tumor diagnostic experience independently utilized ITK-Snap software (version 4.0) to delineate the tumor boundaries and create the region of interest (ROI) in a blinded manner. Intra- and inter-observer reproducibility were evaluated using the intraclass correlation coefficient (ICC) to ensure that the selected features were not influenced by segmentation uncertainties.

Our study employed various essential techniques to address significant challenges in medical image analysis. The CT pixel values were restricted to a range of −125 to 225 HU (Hounsfield Units) to standardize the dataset and mitigate the influence of extremes. For the feature extraction, we applied absolute resampling with a fixed bin width of 5 HU, resulting in a total of 70 bins across the intensity range. This approach ensures consistent quantization across all the patients regardless of intensity distribution differences.

For spatial normalization, we employed fixed-resolution resampling to address voxel spacing inconsistencies in different ROIs. This absolute resampling approach, rather than relative resampling, was chosen to ensure standardized spatial resolution for all ROIs regardless of the original acquisition parameters, achieving uniform voxel spacing of 1 mm × 1 mm × 1 mm across all the images.

### 2.4. Habitat-Based Radiomics Procedure

#### 2.4.1. Delineation of Habitat Subregions

**Figure 2:**
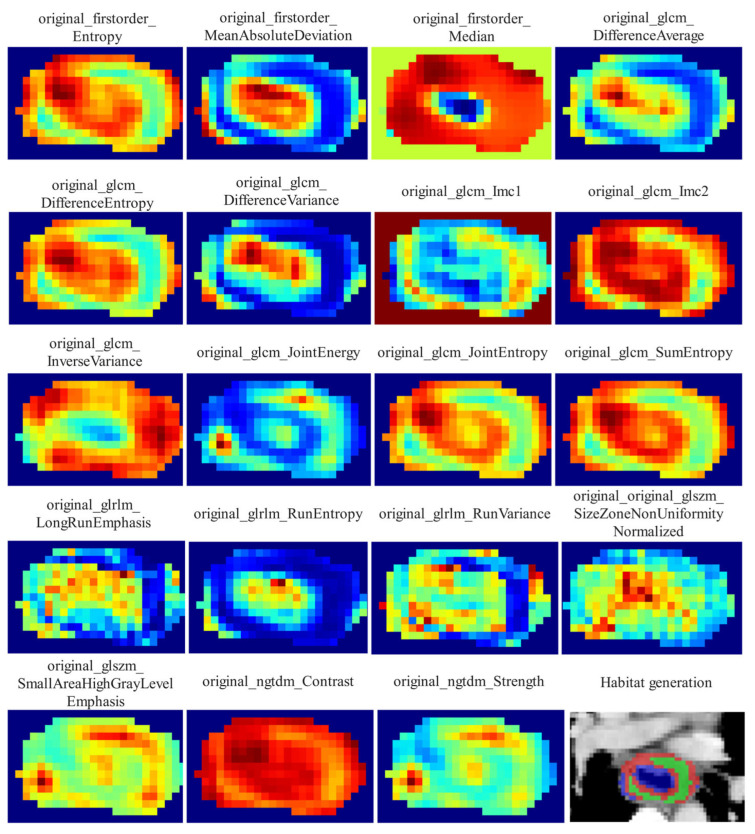
Local features extracted for clustering and the resulting habitat regions.

Local features, such as local entropy and energy values, were extracted from each voxel within the ROIs using the OKT-gen_roi_rad_features tool. These features were amalgamated to form feature vectors encapsulating various attributes of each voxel’s characteristics. To calculate the local features for each voxel, a 3 × 3 × 3 sliding window was employed, enabling the extraction of 19 distinct feature vectors per voxel. These feature vectors were then subjected to K-means clustering to identify subregions within the tumor. Voxels exhibiting similar characteristics were grouped together, with each voxel assigned to one of the resulting clusters and spatially mapped as a habitat within the original image. A pre-determined three-cluster configuration was adopted, informed by existing habitat-related studies to prevent excessive parameter tuning [22]. Details of the habitat generation process and the specific features used are illustrated in Figure 2.

#### 2.4.2. Feature Extraction

From each tumor subregion, a total of 1834 handcrafted radiomic features were extracted from portal venous-phase CT images and categorized into geometry (14 features), intensity (360 features), and texture (1460 features) categories. The geometry features encompassed the three-dimensional shape characteristics of the tumor, while the intensity features described the statistical distribution of voxel intensities within the tumor using first-order analysis. The texture features captured patterns and spatial distributions of intensities using second- and higher-order analysis. Various methods, including the gray-level co-occurrence matrix (GLCM), gray-level dependence matrix (GLDM), gray-level run length matrix (GLRLM), gray-level size zone matrix (GLSZM), and neighborhood gray-tone difference matrix (NGTDM), were utilized to extract the texture features. Since the clustering algorithm employed was unsupervised, it was not guaranteed that each subregion had the same label after clustering. To resolve this issue, we calculated the mean of the features for each subregion to represent the final attributes. For each patient, 1834 radiomic features were also extracted from the entire tumor ROI for comparison. The feature extraction process was carried out using an in-house program implemented in Pyradiomics 3.0.1 (http://pyradiomics.readthedocs.io, accessed on 11 May 2024).

#### 2.4.3. Feature Selection

To assess the robustness of the extracted image features from the ROIs, we conductedtest–retest and inter-rater analyses to ensure that the selected features were not influenced by segmentation uncertainties. The test–retest analysis involved one radiologist performing two segmentations at two-month intervals on each of the randomly selected 30 patients, while the inter-rater analysis required two radiologists to independently segment the ROIs for a separate set of 30 randomly selected patients. The features extracted from the segmented regions were assessed using the intraclass correlation coefficient (ICC), with those exhibiting an ICC ≥ 0.85 considered robust against segmentation uncertainties. After initial screening using the ICC, all the features were standardized using Z-scores to ensure a normal distribution. Subsequently, the *p*-values for all the imaging features were calculated using a *t*-test, retaining only radiomic features with a *p*-value < 0.05. Highly repeatable features were further analyzed using Pearson’s correlation coefficient to identify strongly correlated features. In cases where the correlation coefficient between any two features exceeded 0.9, only one feature was retained. To preserve the maximum feature representation ability, we implemented a greedy recursive deletion strategy to filter the features, removing the feature with the highest redundancy from the current set at each step. The final set of features used to create the radiomic signature was selected through the least absolute shrinkage and selection operator (LASSO) regression model. LASSO regression shrinks regression coefficients towards zero, effectively setting many irrelevant features’ coefficients to zero, based on the regularization weight λ. To identify the optimal λ for the LASSO regression, the 10-fold cross-validation approach was employed. The λ yielding the smallest mean squared error (MSE) between the predicted and actual LNM across the validations was chosen to select the final features.

#### 2.4.4. Development of Two Handcrafted Radiomic Signatures

In this study, we compared the performance of different approaches to tumor region analysis for lymph node metastasis (LNM) prediction: analyzing the tumor region as a whole (Intra) and assessing tumor habitat (Habitat). For the intra radiomic signature (Intra_Rad), we applied Lasso feature selection followed by various machine learning methods to derive the radiomic signature. Specifically, we utilized widely adopted machine learning models, including logistic regression (LR) for linear classification, Support Vector Machines (SVMs), Random Forest, Extra Trees, Extreme Gradient Boosting (XGBoost), and Light Gradient Boosting Machine (LightGBM) for tree-based algorithms and Multi-Layer Perceptron for deep learning, to construct our risk model. In contrast, the habitat signature (Habitat_Rad) was developed through unsupervised clustering algorithms, which limited our ability to ascertain that clusters sharing the same centers represented similar physical meanings. To address this issue, we computed the mean values of the features. Furthermore, due to the unsupervised nature of the clustering, the feature selection process for the habitat signature did not incorporate the ICC evaluation; however, all the other configurations were aligned with those of the Intra models.

### 2.5. 2.5D Deep Learning Procedure

#### 2.5.1. 2.5D Data Generation

To balance the advantages of 2D and 3D approaches, we employed a 2.5D methodology that incorporates spatial context while maintaining computational efficiency. For each patient, we first identified the CT slice with the largest cross-sectional area of the tumor ROI. Instead of using only immediately adjacent slices, we extracted the central slice together with two slices located two layers above and two layers below the central slice (i.e., at positions ±2 and ±4 slices from the central slice). This resulted in a stack of five slices per patient: the central slice, the slices at ±2 layers, and the slices at ±4 layers. By introducing this interval-based selection, we partially preserved 3D structural information while reducing data redundancy and computational demand. This process was implemented using OKT-crop_max_roi with the parameter surrounds of +2, +4, −2, and −4.

#### 2.5.2. Model Training

All the generated 2.5D data were incorporated into a transfer learning framework. Instead of merging the slices into a single planar image, each slice was independently processed by convolutional neural network (CNN) models, specifically DenseNet201, ResNet50, and VGG19, all of which were pre-trained on the ImageNet Large Scale Visual Recognition Challenge 2012 (ILSVRC2012) dataset. Prior to inputting into the networks, each 2D slice underwent preprocessing that involved normalizing the gray values to the range [−1, 1] using min–max normalization and resizing to 224 × 224 pixels via nearest-neighbor interpolation, in order to meet the input requirements of the pre-trained models. Stochastic Gradient Descent (SGD) was employed as the optimizer, and sigmoid cross-entropy was utilized as the loss function. Due to the limited size of the image dataset, particular care was taken in selecting an appropriate learning rate to improve model generalization. In this study, we adopted the cosine decay learning rate schedule. The specific learning rate settings used in our experiments are detailed in Appendix A.3.

The model training was performed on a workstation equipped with a Windows 10 operating system, an Intel Core i9-14900KF processor, 96 GB of DDR5 RAM, and an NVIDIA (Santa Clara, CA, USA) GeForce RTX 4090 GPU with 24 GB of VRAM. Under this hardware configuration, the training of each model required approximately 2 h.

#### 2.5.3. Multi-Instance Learning

**Figure 3:**
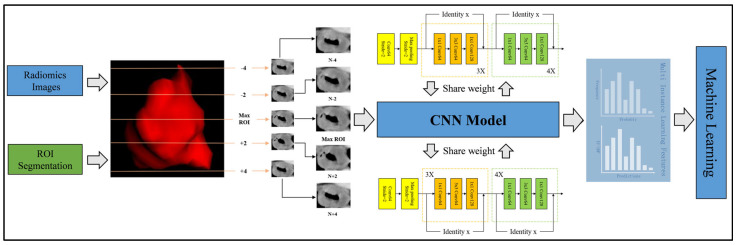
Comprehensive workflow of the 2.5D deep learning and multiple instance learning processes. The diagram illustrates the extraction of the maximum cross−sectional slice and adjacent slices, followed by individual processing through convolutional neural network (CNN) architectures and subsequent feature integration using multiple instance learning techniques.

To address the predictions from the deep learning models, we introduced two fusion methods for multi-instance learning (MIL), akin to those in pathological image analysis [23]. The first method, the predict likelihood histogram (PLH), utilizes 2.5D deep learning models to generate predictive probabilities and labels for cross-sectional areas of 2.5D images. By expanding the use of PLH channels, we created a histogram of probability distributions that accurately represented the image features, offering a detailed image portrayal. The second method, bag of words (BoW), segments the full image into slices to extract probabilities and predictions from each, combining 2.5D and multi-model results to yield 3 × 5 predictive outcomes (3 models, 5 slices) per sample. Mirroring the BoW approach from textual analysis, we treated these predictive outcomes as features similar to word frequencies in text, utilizing TF-IDF (Term Frequency–Inverse Document Frequency) for the feature characterization. By integrating the feature representations from the PLH and BoW, we forged a comprehensive feature set from MIL, merging various information sources for an adept depiction of image characteristics. The process of multi-instance learning feature fusion is detailed in Appendix A.4. As shown in Figure A4, the feature selection method and process after fusion are consistent with the feature selection steps of habitat-based radiomics. Figure 3 visually depicts the comprehensive workflow of the 2.5D deep learning and multiple instance learning process.

#### 2.5.4. Construction of a Multi-Instance Learning Radiomic Signature

The selected features from MIL were then inputted into machine learning algorithms akin to those used for radiomic feature modeling to construct the MIL radiomic signature (MIL_Rad). During the model training process, we similarly employed 5-fold cross-validation within the training set, combined with Grid-Search for hyperparameter optimization.

### 2.6. Building a Clinicoradiological Signature and Nomogram

In the training cohort, significant clinical and radiologic predictors were identified via univariate and multivariate logistic regression, with odds ratios (ORs) and 95% confidence intervals (CIs) calculated. A clinicoradiological signature was developed as a baseline. A nomogram was then created by combining the Habitat_Rad and MIL_Rad signatures with independent predictors to further improve performance.

### 2.7. Model Performance Assessment and Interpretability

The performance of each model in predicting LNM was evaluated by calculating the area under the curve (AUC) of the receiver operating characteristic (ROC) curve. Furthermore, the corresponding metrics—accuracy, sensitivity, specificity, positive predictive value (PPV), and negative predictive value (NPV)—were computed. Calibration of all models was assessed in both the training and test groups using calibration curves derived from 1000 resampling bootstraps, as well as the Hosmer–Lemeshow goodness-of-fit test. Decision curve analysis (DCA) was conducted to estimate the clinical utility of each model by quantifying the net benefit across various threshold probabilities.

### 2.8. Statistics

The statistical analyses were conducted using SPSS (version 26.0, IBM) and Python (version 3.8; http://www.python.org, accessed on 11 May 2024). The continuous variables were compared using the Student’s *t*-test or the Mann–Whitney U-test, while the categorical variables were analyzed with the chi-square test or Fisher’s exact test, as appropriate. The area under the curves (AUCs) for different models were compared using the DeLong test. Univariate and multivariate Cox proportional hazards regression analyses were performed to identify independent predictors of lymph node metastasis (LNM). A two-tailed *p*-value of less than 0.05 was considered statistically significant.

## 3. Results

### 3.1. Clinicoradiological Signature

The baseline clinicoradiological characteristics of 469 ESCC patients are summarized in Table A1. LNM was observed in 98 (29.9%) patients in the training cohort and 39 (27.7%) patients in the test cohort. Multivariate logistic regression analysis demonstrated that LNM was less likely to occur in older patients (OR = 0.992, 95% CI: 0.986–0.998, *p* = 0.028) and with treatment from NACRT (OR = 0.889, 95% CI: 0.819–0.966, *p* = 0.019) but more likely to be associated with a larger MLNSD (OR = 1.013, 95% CI: 1.004–1.022, *p* = 0.022) (Table 1). These three independent predictors form a clinicoradiological signature used to develop LNM prediction models.

The LightGBM-based model demonstrated superior performance in the independent test cohort with an AUC of 0.716 (95% CI: 0.587–0.845) (Table 2). The detailed results from the five-fold cross-validation in the training cohort are provided in Table A2.

### 3.2. Performance of Habitat-Based Radiomic Signature

**Figure 4:**
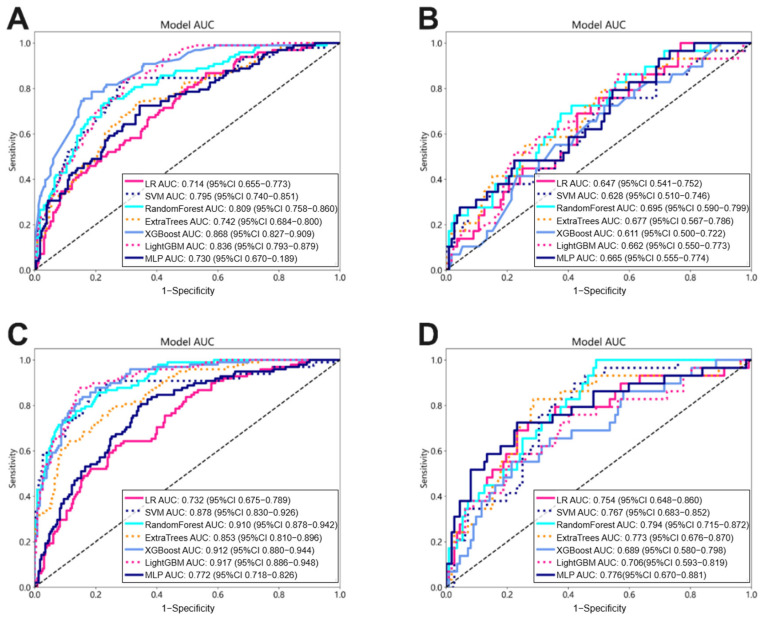
Performance of two types of handcrafted signatures. Receiver operating characteristic (ROC) curves of the Intra_Rad signature on the training (**A**) and testing (**B**) datasets, and the Habitat_Rad signature on the training (**C**) and test (**D**) sets.

**Figure A2:**
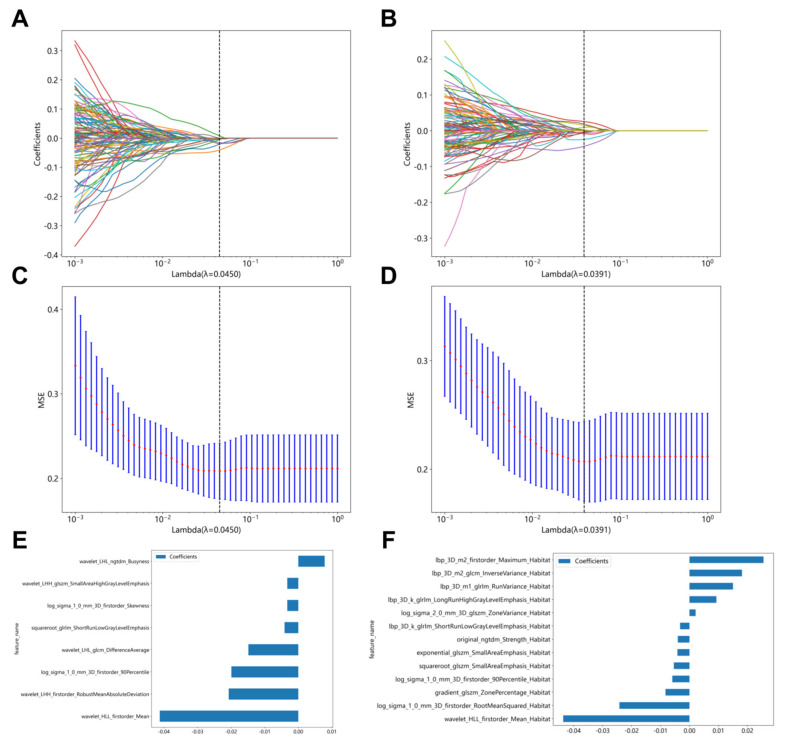
Feature selection with the least absolute shrinkage and selection operator (LASSO) algorithm. (**A**,**C**,**E**) represents the whole-tumor-based radiomic signature (Intra_Rad), and (**B**,**D**,**F**) represents the subregion-based radiomic signature (Habitat_Rad). (**A**,**B**) Variation curve of the regression coefficient with λ. (**C**,**D**) The cross-validation curve. The dotted vertical lines were drawn at the optimal λ value by using 10-fold cross-validation and the mean standard error (MSE) of the minimum criteria. (**E**,**F**) Weight visualization of features after LASSO selection.

Eight and thirteen features were selected to build the Intra_Rad and Habitat_Rad, respectively (Figure A2). As shown in Figure 4, the Habitat_Rad achieved a better predictive performance than the corresponding Intra_Rad with an improvement of nearly 0.1 in the AUC values. Specifically, within the top models constructed by Random Forest, the Habitat_Rad signature demonstrated AUCs of 0.910 (95% CI: 0.880–0.944) and 0.794 (95% CI: 0.715–0.872) on the training and test cohorts, compared to AUCs of 0.809 (95% CI: 0.758–0.860) and 0.695 (95% CI: 0.590–0.799) for the Intra_Rad on the training and testing cohorts, respectively.

### 3.3. Performance of DL Models and MIL_Rad Signature

**Figure A3:**
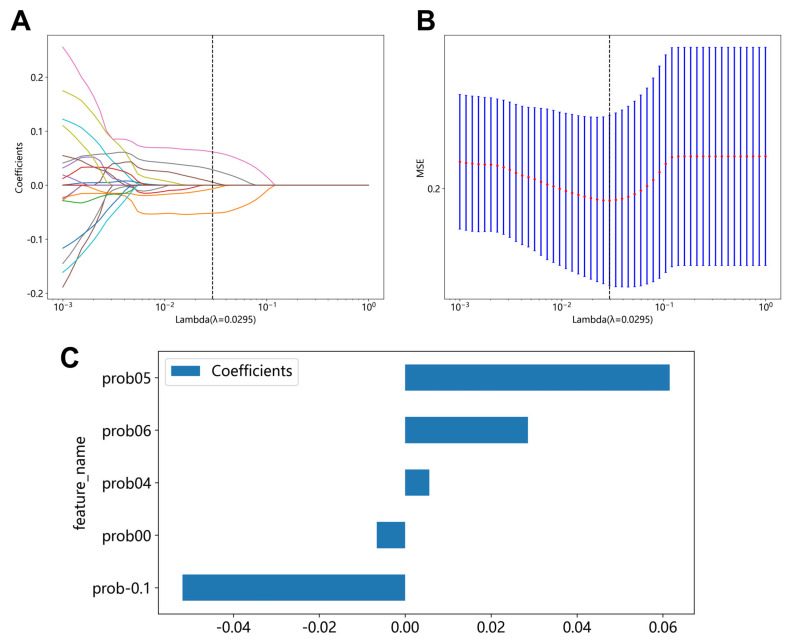
The process of feature selection for constructing a multi-instance learning radiomic signature. (**A**) Variation curve of the regression coefficient with λ. (**B**) The cross-validation curve. The dotted vertical lines were drawn at the optimal λ value by using 10-fold cross-validation and the mean standard error (MSE) of the minimum criteria. (**C**) Weight visualization of features after LASSO selection.

Five key features were selected to construct the MIL_Rad signature (Figure A3). While individual 2.5D deep learning models demonstrated limited discriminative ability with AUC values below 0.7 in the test cohort, the MIL-Rad signature showed significantly enhanced performance. Specifically, the signature developed using the Extra Trees algorithm achieved an AUC of 0.796 (95% CI: 0.688–0.904) in the test set, surpassing the performance of the other modeling approaches (Table 3). The comprehensive results from the five-fold cross-validation in the training cohort are available in Table A3.

### 3.4. Fusion Nomogram for Clinical Use

**Figure 5:**
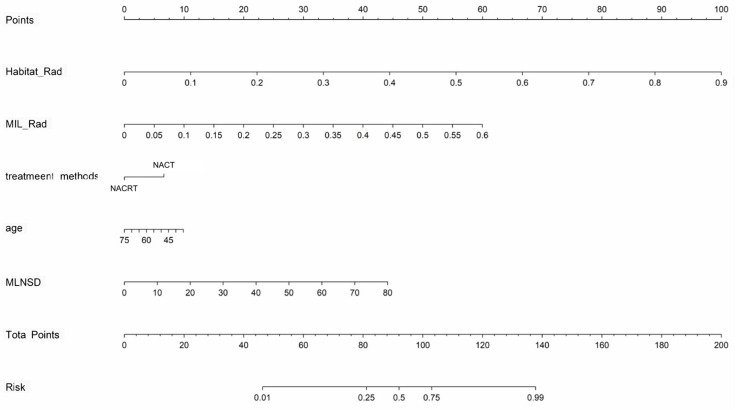
Nomogram constructed based on clinicoradiological factors, the habitat_Rad signature, and the MIL_Rad signature for predicting lymph node metastasis. MLNSD, maximum lymph node short diameter; NACRT, neoadjuvant chemoradiotherapy; and NACT, neoadjuvant chemotherapy.

The best-performing models in the independent test cohort was selected from the Habitat-Based radiomic signature and the MIL_Rad signature and subsequently integrated with independent clinicoradiological predictors using logistic regression to construct a late-fusion model. A predictive nomogram was then developed by incorporating clinicoradiological factors, the Habitat_Rad signature, and the MIL_Rad signature to predict LNM in ESCC patients undergoing neoadjuvant therapy. The nomogram, which facilitates clinical utilization, is illustrated in Figure 5. A detailed description of the construction and application of the nomogram can be found in Appendix A.5.

### 3.5. Performance Comparison Among Various Signatures

**Figure 6:**
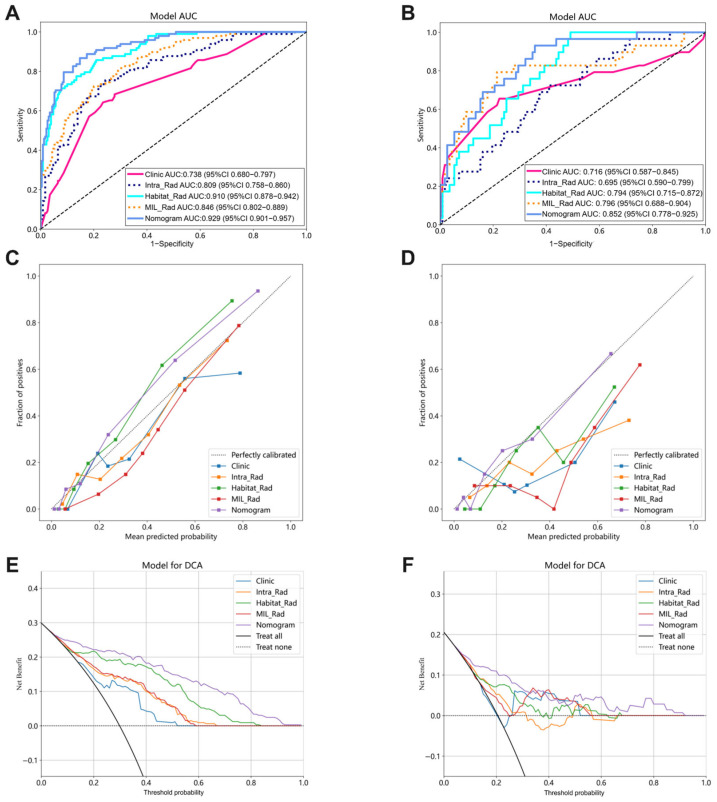
Performance of various models. Receiver operating characteristic (ROC) curves of the training (**A**) and test (**B**) sets; calibration curves in the training (**C**) and test (**D**) datasets; and decision curve analysis (DCA) of the training (**E**) and test (**F**) groups.

**Figure A4:**
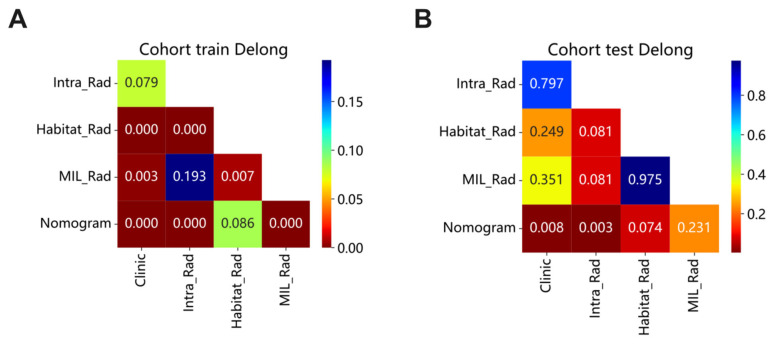
*p*-values of the DeLong test comparing ROC curves of different models for the training set (**A**) and test set (**B**).

The best-performing model in the test cohort from each signature, together with the late-fusion nomogram model, was included for performance comparison. As shown in Figure 6A,B, the nomogram achieved an AUC of 0.929 (95% CI: 0.901–0.957) in the training set and 0.852 (95% CI: 0.778–0.925) in the test set, indicating robust discriminative ability for assessing LN status after NAT in ESCC patients. Pairwise comparisons of the AUCs using the non-parametric DeLong test confirmed that the nomogram significantly outperformed both the Clinic and Intra_Rad signatures (*p* < 0.05, Figure A4). In addition, the Hosmer–Lemeshow test produced *p*-values of 0.926 for the training cohort and 0.666 for the test cohort, indicating a good calibration of the model (Figure 6C,D). Furthermore, decision curve analysis (DCA) demonstrated that the nomogram provides a substantial net benefit across a range of predicted probabilities and outperforms the other signatures in terms of clinical utility (Figure 6E,F).

## 4. Discussion

In this study, we developed several predictive models using pre-treatment contrast-enhanced CT images and clinicoradiological factors to assess the status of lymph node metastasis (LNM) at the time of surgery in patients with esophageal squamous cell carcinoma (ESCC) following neoadjuvant therapy (NAT). Among these, the combined nomogram model, integrating a tumor-habitat-based radiomic signature, a multiple instance learning (MIL)-based signature derived from 2.5D deep learning models, and independent clinicoradiological risk factors, exhibited superior performance.

NAT for locally advanced ESCC can significantly reduce tumor staging before surgery and increase the rate of complete resection [24,25]. LNM status is a crucial prognostic factor in esophageal cancer and plays a significant role in determining personalized perioperative treatment strategies [26]. To address the limitations of current radiological methods in preoperative assessment of LNM [27,28], our study developed and validated a series of models, including those based on clinicoradiological factors, handcrafted radiomic features, 2.5D deep learning, and combined approaches.

In terms of clinicoradiological factors, univariable and multivariable analyses identified two clinical characteristics (age and treatment method) and one peripheral LN radiographic feature (maximum lymph node short diameter) as independent risk factors, which aligns with previous studies’ findings [29,30,31]. The risk factor-based models demonstrated inadequate discriminative capabilities, with the top model constructed by LightGBM achieving an AUC of 0.738 on the training dataset and 0.716 on the test set, indicating limited effectiveness in predicting LNM.

Our study demonstrated that tumor-habitat-based radiomics (Habitat_Rad) significantly outperformed whole-tumor-based approaches (Intra_Rad) in predicting LNM in ESCC. This aligns with previous findings showing similar superiority in intrahepatic cholangiocarcinoma [32], breast cancer [33], and cervical cancer [34]. Tumor subregions, characterized by distinct tissue structures and functional properties [35], arise from heterogeneity in the vasculature, metabolism, and gene expression during tumor progression [36,37]. CT imaging reveals these subregions through variations in density, morphology, and texture [38], reflecting biological features such as necrosis, hemorrhage, calcification, and cellular proliferation. Therefore, exploring the relationships between imaging features of tumor subregions and LNM is crucial for improving tumor diagnosis, optimizing treatment strategies, and enhancing prognostic evaluations.

A single slice only provides information in the transverse plane, which means 3D anatomical information is lost during the training process, resulting in unreliable outcomes. A study on the performance of multi-organ cancer classification based on 2D and 3D image features in radiomic analysis shows that in several aspects, including LNM prediction, 3D image features provide predictive performance that is superior to or equal to 2D image features [39]. However, 3D deep convolutional neural networks (DCNNs) often require significantly more parameters to train, and limited data and high computational cost typically hinder their performance. Therefore, we proposed a 2.5D method to convert 3D data into 2D images by integrating the largest tumor layer with the two layers above and below and then training it on 2D DCNNs. On the one hand, the 2.5D model captures more contextual information and is more effective than a pure 2D model. At the same time, it requires less computation than a 3D model, offering a balanced solution for performance and efficiency. To address the unsatisfactory performance of individual 2.5D deep learning (DL) models, we employed the multiple instance learning (MIL) method for model fusion, which led to more comprehensive representation. The resulting MIL-based signature demonstrated significantly improved performance compared to single 2.5D models.

In our study, the combined nomogram showed superior performance with AUC values of 0.929 in the training cohort and 0.852 in the test cohort. Tan et al. constructed a nomogram integrating radiomic features with CT-reported LN status for LNM prediction in resectable ESCC patients, achieving AUC values of 0.758 and 0.773 in the training and test sets, significantly outperforming traditional size criteria (AUCs of 0.661 and 0.586, respectively) [40]. Wu et al.’s multi-level CT radiomic model, designed to preoperatively forecast LNM in ESCC, exhibited AUCs of 0.875 in the training cohort and 0.874 in the internal validation cohort. By incorporating clinical variables alongside handcrafted, computer vision (CV), and deep learning signatures, this model outperformed Model 1 (clinical predictors and handcrafted signature) and Model 2 (clinical predictors plus handcrafted and CV signatures) [41]. These studies highlight the crucial role of radiomics in capturing tumor heterogeneity of ESCC and indicate the performance boost from combining multiple radiomic signatures.

Our study encountered several limitations. Firstly, although our model performs well on both the training and testing sets, it still faces the risk of overfitting and limited generalizability. In response to concerns about generalizability with a single train–test split, we implemented 5-fold cross-validation during the model development, which improved the robustness of our findings. However, we acknowledge that external validation with independent cohorts from different institutions remains necessary to fully establish the generalizability of our model. Secondly, the reproducibility of habitat division is a challenge. Unsupervised learning methods based on K-means clustering may introduce variability due to differences in equipment, scanning parameters, and image preprocessing steps. We have attempted to alleviate this issue through strict image standardization and feature selection processes, but future research should explore more robust habitat identification algorithms and standardization procedures. Thirdly, due to the relatively small dataset, our study primarily focused on binary classification predictions (presence/absence of LNM) without distinguishing LNM patterns in different regions (neck, chest, and abdomen), which is crucial for individualized treatment planning. Future studies should consider developing multi-class prediction models to assess the risk of lymph node metastasis in specific anatomical regions. Finally, the ethical issues of AI-assisted clinical decision-making systems cannot be ignored. AI-based predictions may lead to over-reliance or misinterpretation, especially when clinicians find it difficult to explain the model’s decision logic. We emphasize that the model proposed in this study should serve as an auxiliary tool for clinical decision-making rather than a replacement for clinical judgment. Future research and applications should prioritize model transparency, interpretability, and continuous monitoring of patient outcomes.

## 5. Conclusions

This study developed and validated a CT-based radiomic nomogram that integrates clinicoradiological features, a tumor-habitat-based radiomic signature, and deep learning models, demonstrating excellent discrimination ability in predicting LNM status in ESCC patients after NAT. As a non-invasive preoperative approach, the radiomic nomogram could assist in clinical decision-making and potentially improve outcomes for ESCC patients.

## Figures and Tables

**Table 1:** Univariable and multivariable analyses for selecting clinicoradiological features in the training cohort.

Variable	Univariable		Multivariable	
	OR (95% CI)	*p*-Value	OR (95% CI)	*p*-Value
Age (year), median (IQR)	0.992 (0.986–0.998)	0.038	0.992 (0.986–0.998)	0.028
Sex	0.899 (0.805–1.003)	0.110		
Smoking history	1.062 (0.974–1.157)	0.251		
Drinking history	1.134 (1.044–1.232)	0.013	1.086 (1.000–1.178)	0.098
cT stage	0.929 (0.865–0.997)	0.084		
cN stage	1.090 (1.0441.139)	0.001	1.032 (0.984–1.082)	0.275
Treatment methods	0.863 (0.794–0.938)	0.004	0.889 (0.819–0.966)	0.019
TMD (mm), median (IQR)	1.001 (0.996–1.005)	0.835		
Tumor enhancement pattern	1.017 (0.972–1.064)	0.540		
MLNSD (mm), median (IQR)	1.018 (1.011–1.025)	<0.001	1.013 (1.004–1.022)	0.022
MLNF	0.995 (0.968–1.024)	0.776		
MLNEV	1.006 (0.979–1.035)	0.702		
MLNN	1.015 (0.988–1.042)	0.369		

TMD, tumor maximum diameter; MLNSD, maximum lymph node short diameter; MLNF, maximum lymph node fusion; MLNEV, maximum lymph node extracapsular violation; and MLNN, maximum lymph node necrosis.

**Table 2:** Performance of the clinicoradiological signature (Clinic) in the independent test cohort.

Model	Accuracy (95% CI)	AUC (95% CI)	Sensitivity (95% CI)	Specificity (95% CI)	PPV	NPV
LR	0.787 (0.743–0.832)	0.708 (0.579–0.837)	0.586 (0.533–0.640)	0.839 (0.800–0.879)	0.486	0.887
SVM	0.780 (0.735–0.825)	0.702 (0.571–0.833)	0.621 (0.568–0.673)	0.821 (0.780–0.863)	0.474	0.893
Random Forest	0.844 (0.805–0.883)	0.714 (0.587–0.840)	0.586 (0.533–0.640)	0.911 (0.880–0.942)	0.630	0.895
Extra Trees	0.787 (0.743–0.832)	0.714 (0.588–0.840)	0.586 (0.533–0.640)	0.839 (0.800–0.879)	0.486	0.887
XGBoost	0.837 (0.797–0.877)	0.713 (0.584–0.842)	0.552 (0.498–0.606)	0.911 (0.880–0.942)	0.615	0.887
LightGBM	0.752 (0.705–0.799)	0.716 (0.587–0.845)	0.655 (0.604–0.707)	0.777 (0.732–0.822)	0.432	0.897
MLP	0.794 (0.751–0.838)	0.695 (0.562–0.828)	0.621 (0.568–0.673)	0.839 (0.800–0.879)	0.895	0.500

**Table 3:** Performance of individual 2.5D deep learning (DL) models and multiple instance learning-based signature (MIL_Rad) in the independent test set.

Model	Accuracy (95% CI)	AUC (95% CI)	Sensitivity (95% CI)	Specificity (95% CI)	PPV	NPV
**Individual 2.5D** **DL** **model**						
ResNet50	0.564 (0.519–0.609)	0.620 (0.573–0.667)	0.697 (0.656–0.739)	0.526 (0.480–0.571)	0.298	0.857
DenseNet201	0.236 (0.197–0.274)	0.625 (0.578–0.672)	1.000 (1.000–1.000)	0.015 (0.004–0.026)	0.227	1.000
VGG19	0.667 (0.703–0.782)	0.607 (0.561–0.654)	0.280 (0.239–0.321)	0.779 (0.936–0.974)	0.268	0.789
**MIL_Rad**						
LR	0.801 (0.758–0.845)	0.728 (0.615–0.840)	0.586 (0.533–0.640)	0.865 (0.819–0.895)	0.515	0.889
SVM	0.830 (0.789–0.870)	0.724 (0.602–0.847)	0.483 (0.429–0.537)	0.920 (0.890–0.949)	0.609	0.873
Random Forest	0.858 (0.820–0.896)	0.787 (0.679–0.895)	0.586 (0.533–0.640)	0.929 (0.901–0.956)	0.680	0.897
Extra Trees	0.787 (0.743–0.832)	0.796 (0.688–0.905)	0.793 (0.749–0.837)	0.786 (0.741–0.830)	0.489	0.936
XGBoost	0.652 (0.601–0.704)	0.728 (0.626–0.830)	0.828 (0.787–0.868)	0.607 (0.554–0.660)	0.353	0.932
LightGBM	0.667 (0.616–0.718)	0.737 (0.631–0.844)	0.793 (0.749–0.837)	0.645 (0.582–0.686)	0.359	0.922
MLP	0.809 (0.766–0.851)	0.717 (0.603–0.830)	0.552 (0.498–0.606)	0.875 (0.839–0.911)	0.533	0.883

LR, logistic regression; SVM, Support Vector Machines; XGBoost, Extreme Gradient Boosting; LightGBM, Light Gradient Boosting Machine; MLP, Multi-Layer Perceptron; AUC, area under the curve; PPV, positive predictive value; and NPV, negative predictive value.

## Data Availability

The datasets of this research are backed up on the Research Data Deposit (RDD, https://www.researchdata.org.cn, accessed on 11 December 2024, approval number: RDDA20240402) and are available on reasonable request.

## Abbreviations

The following abbreviations are used in this manuscript:

DL	Deep Learning
EC	Esophageal Carcinoma
ESCC	Esophageal Squamous Cell Carcinoma
LNM	Lymph Node Metastasis
MIL	Multiple Instance Learning
NAT	Neoadjuvant Therapy
NACT	Neoadjuvant Chemotherapy
NACRT	Neoadjuvant Chemoradiotherapy
ROI	Region of Interest

## Appendix A

### Appendix A.1. Neoadjuvant Chemotherapy Regimens

It is well known that a standardized treatment regimen for esophageal squamous cell carcinoma (NAT) has yet to be established; this retrospective study only included patients receiving the NAT regimens recommended by the National Comprehensive Cancer Network (NCCN). NAT regimens are classified as follows: (1) a radiotherapy regimen, which utilizes intensity-modulated radiation therapy. After acquiring the patients’ CT images, they were uploaded into the radiotherapy planning system for target delineation and plan design. Based on ICRU Report 62 and our center’s treatment experience, the target delineation is defined as follows: the gross tumor target includes the primary esophageal tumor and metastatic lymph nodes. The primary lesion is delineated by identifying the areas where the esophageal wall thickness exceeds 5 mm in the portal venous phase of CT-enhanced scans, with the tumor boundaries determined by combining the findings from barium swallow studies and endoscopic ultrasound, with PET-CT used as a reference when available; metastatic lymph nodes include those with a short-axis greater than 10 mm on CT or those that demonstrate high metabolic activity on PET-CT. The clinical tumor target comprises a 0.8–1.0 cm margin around the gross tumor target and at least 3 cm of normal esophageal tissue in the craniocaudal direction, as well as the lymphatic drainage area of positive lymph nodes, which includes at least a 1–1.5 cm margin around the metastatic lymph nodes, adjusted appropriately for anatomical barriers. In principle, prophylactic irradiation is not performed on regions of negative lymph nodes in accordance with the involved-field irradiation concept. The planning tumor target extends 0.5–1 cm beyond the clinical tumor target. To ensure sufficient dose delivery to the tumor, the 95% isodose curve must cover 99% of the planning tumor target, while the important organs at risk—the heart, lungs, and spinal cord—are kept to minimal doses (bilateral lung V20 ≤ 35%, V5 ≤ 65%; heart V30 ≤ 40%, V40 ≤ 30%; and maximum spinal cord dose < 50 Gy). The prescribed radiotherapy dose is 44 Gy to the primary tumor and metastatic lymph nodes and 40 Gy to the upper and lower 3 cm of normal esophageal tissue and the corresponding lymph node regions, delivered in 20 fractions over 3–4 weeks (five fractions per week, one fraction per day). (2) There is a chemotherapy regimen, where 60.5% of patients received concurrent chemotherapy and 39.5% received single-agent chemotherapy. All patients were treated with platinum-based chemotherapy. The chemotherapy drugs primarily included paclitaxel, cisplatin, nedaplatin, and 5-fluorouracil. Among the double-drug regimens, the DP regimen consisted of docetaxel and cisplatin, the TP regimen of paclitaxel and nedaplatin, and the NP regimen of vinorelbine and cisplatin; the three-drug regimen was the TPF regimen, which included docetaxel, cisplatin, and 5-fluorouracil. All patients followed the standard course of NAT regimens.

### Appendix A.2. Surgical Approach

The surgical techniques implemented comprised right thoracic and upper abdominal two-incision partial esophagectomy, left thoracic partial esophagectomy, and a three-incision partial esophagectomy involving the left cervical, right thoracic, and upper abdominal regions. All the procedures were conducted with contemporary two-field lymphadenectomy, which necessitated the dissection of both thoracic and upper abdominal lymph nodes. For patients with suspected cervical lymph node metastasis, cervical lymphadenectomy was additionally performed. Following the resection of the esophageal lesion, the surgeon categorized the lymph nodes in the specimen, designating and numbering them according to the Japanese regional lymph node classification prior to submission to the pathologist.

### Appendix A.3. Learning Rate Settings for 2.5D Deep Learning Model Training

Given the limited availability of image data, we carefully determined the learning rate to enhance generalization. In this study, we employed the cosine decay learning rate algorithm. The specific learning rate used in our experiments is presented in Appendix A.3 as follows:

(A1)
$$\eta_{t}^{task-spec}=\eta_{min}^{i}+\frac{1}{2}\left(\eta_{max}^{i}-\eta_{min}^{i}\right)\left(1+cos\left(\frac{T_{cur}}{T_{i}}\pi \right)\right)$$

The minimum learning rate, denoted as $\eta_{min}^{i}$ , is set to 0, while the maximum learning rate, denoted as $\eta_{max}^{i}$ , is set to 0.01. The parameter $T_{i}$ represents the number of iteration epochs. Since the backbone part of the model utilizes pre-trained parameters, we performed fine-tuning on the backbone part at $T_{cur}=\frac{1}{2}T_{i}$ to ensure effective transfer of knowledge. Consequently, the learning rate for the backbone part is determined as follows:

(A2)
$$\eta_{t}^{backbone}=\left\{\begin{array}{ll} 0 & \mathrm{if}\;T_{cur}\leq \frac{1}{2}T_{i}\\ \eta_{min}^{i}+\frac{1}{2}\left(\eta_{max}^{i}-\eta_{min}^{i}\right)\left(1+cos\left(\frac{T_{cur}}{T_{i}}\pi \right)\right) & \mathrm{if}\;T_{cur}>\frac{1}{2}T_{i} \end{array}\right.$$

### Appendix A.4. Multi-Instance Learning-Based Feature Fusion

In our study, we implemented two multi-instance learning fusion techniques. Using 2.5D deep learning models, we created predict likelihood histograms (PLHs) that map out the predictive probabilities and labels for each slice, offering a probabilistic summary of the prediction landscape. We also applied a bag of words (BoW) approach, cropping each entire 3D volume into five slices and extracting data using three DL models to compile 15 predictive results per sample, using the Term Frequency–Inverse Document Frequency (TF-IDF) method for analysis. Additionally, we enhanced our model by integrating PLH and BoW features with radiomic data, leveraging diverse data sources to improve the representational power and accuracy of our classification tasks.

Our multi-instance learning approach aimed to enhance predictive accuracy by integrating various data points from a single sample into a comprehensive feature set, involving the following:

1. Slice Prediction: Each slice was analyzed using the deep learning model to derive probabilities and labels, denoted as Slice_prob_ and Slice_pred_, retained to two decimal places.
2. Multi-instance Learning Feature Aggregation
  2.1 Histogram Feature Aggregation:
    ▪ Distinct numbers were treated as “bins” to count occurrences across types.
    ▪ Frequencies of Slice_prob_ and Slice_pred_ in each bin were tallied and normalized using min–max normalization, resulting in Histo_prob_ and Histo_pred_.
  2.2 Bag of Words (BoW) Feature Aggregation:
    ▪ A dictionary was constructed from unique elements in Slice_prob_ and Slice_pred_.
    ▪ Each slice was represented as a vector noting the frequency of each dictionary element, with a TF-IDF transformation applied to emphasize informative features.
    ▪ This resulted in a BoW feature representation for each slice, encapsulating both the presence and significance of features.
3. Feature Early Fusion: We integrated Histo_prob_, Histo_pred_, Bow_prob_, and Bow_pred_ using a feature concatenation method (⊕), combining these into a single comprehensive feature vector:

$$feature_{fusion}=Histo_{prob}⊕Histo_{pred}⊕Bow_{prob}⊕Bow_{pred}$$

For the aggregated multi-instance learning features, we utilized dimensionality reduction techniques such as t-tests, correlation coefficients, and Lasso regularization to refine our feature set. These features were modeled using popular machine learning algorithms. To address sample imbalance, we employed the SMOTE method during the training process. To ensure model robustness, we applied 5-fold cross-validation within the training dataset and optimized the hyperparameters via Grid-Search.

### Appendix A.5. Construction and Use of the Nomogram

Construction and Application of the Nomogram

1. Nomogram Construction

To develop an individualized predictive tool for lymph node metastasis, a nomogram was constructed by integrating selected clinical and radiological variables, as well as the radiomic signatures (habitat_Rad and MIL_Rad) derived from our imaging analyses. The construction process consisted of the following steps:

1.1 Variable Selection:

The candidate clinical and radiological variables were identified based on clinical relevance and statistical significance in univariate and multivariable analysis. The variables showing significant association with lymph node metastasis were further evaluated in a multivariate logistic regression model.

1.2 Logistic Regression Model:

Independent clinicoradiological predictors—including treatment method, age, and maximum lymph node short diameter (MLNSD)—as well as the predicted probability values from the habitat_Rad and MIL_Rad signatures, were incorporated into a multivariable logistic regression model. The regression coefficients derived from this model, reflecting the relative contribution of each variable to the outcome, were then used to assign the points within the nomogram.

1.3 Nomogram Generation:

The logistic regression model was visualized as a nomogram using the “rms” package in R (version 4.3.2). In this graphical tool, each predictor variable is represented as a separate axis. Each value of a variable corresponds to a specific point score, with higher total points reflecting a greater predicted risk of lymph node metastasis.

2. Nomogram Application for Prediction

To predict an individual patient’s probability of lymph node metastasis using the nomogram, the following procedure was applied:

2.1 For each predictor, locate the patient’s value on the corresponding axis of the nomogram. Draw a vertical line to the “Points” axis to determine the point score for each predictor.
  2.2 Sum all the points from each predictor to calculate the total score.
  2.3 Find the total score on the “Total Points” axis and draw a vertical line downward to the “Predicted Probability” axis to obtain the predicted risk of lymph node metastasis for the patient.

## Appendix B. Tables and Figures

**Table A1:** Clinicoradiological variables of patients in the training and test cohorts.

Characteristics	Training Cohort (*n* = 328)			Test Cohort (*n* = 141)		
	LN− (*n* = 230)	LN+ (*n* = 98)	*p*-value	LN− (*n* = 102)	LN+ (*n* = 39)	*p*-Value
Age (year), median (IQR)	60 (53, 64)	59 (51, 62)	0.031	61 (55, 66)	58 (50, 62)	0.170
Sex, *n* (%)			0.149			0.659
Male	185 (80.43)	86 (87.76)		81 (79.41)	30 (76.92)	
Female	45 (19.57)	12 (12.24)		21 (20.59)	9 (23.08)	
Smoking history, *n* (%)			0.304			0.390
No	93 (40.43)	33 (33.67)		46 (45.10)	15 (38.46)	
Yes	137 (59.57)	65 (66.33)		56 (54.90)	24 (61.54)	
Drinking history, *n* (%)			0.018			0.300
No	133 (57.83)	42 (42.86)		52 (50.98)	16 (41.03)	
Yes	97 (42.17)	56 (57.14)		50 (49.02)	23 (58.97)	
cT stage, *n* (%)			0.214			0.279
T2	96 (41.74)	51 (52.04)		48 (47.06)	18 (46.15)	
T3	120 (52.17)	43 (43.88)		47 (46.08)	21 (53.85)	
T4	14 (6.09)	4 (4.08)		7 (6.86)	0	
cNstage			<0.001			0.411
0	69 (30.00)	9 (9.18)		27 (26.47)	6 (15.38)	
1	80 (34.78)	43 (43.88)		43 (42.16)	15 (38.46)	
2	58 (25.22)	32 (32.65)		24 (23.53)	12 (30.77)	
3	23(10.00)	14(14.29)		8 (7.84)	6 (15.38)	
Treatment methods, *n* (%)			0.006			0.005
NACT alone	87 (37.83)	54 (55.10)		38 (37.25)	26 (66.67)	
NACRT	143 (62.17)	44 (44.90)		64 (62.75)	13 (33.33)	
TMD (mm), median (IQR)	32 (30, 40)	33 (30, 42)	0.781	31 (29, 41)	32 (30, 41)	0.220
Tumor enhancement pattern, *n* (%)			0.255			0.430
Homogenous enhancement	45 (19.57)	23 (23.47)		30 (29.41)	8 (20.51)	
Inhomogeneous enhancement	123 (53.48)	54 (55.10)		40 (39.22)	21 (53.85)	
Suspicious enhancement	62 (26.96)	21 (21.43)		32 (30.37)	10 (25.64)	
MLNSD (mm), median (IQR)	8 (6, 11)	12 (9, 17)	<0.001	9 (7, 11)	12 (10, 16)	0.003
MLNF, *n* (%)			0.031			0.022
No	202 (87.83)	77 (78.57)		91 (89.22)	26 (66.67)	
Yes	28 (12.17)	22 (21.43)		11 (10.78)	13 (33.33)	
MLNEV, *n* (%)			0.081			0.528
No	13 6(59.13)	47 (47.96)		54 (52.94)	18 (46.15)	
Yes	94 (40.87)	51 (52.04)		48 (47.06)	21 (53.85)	
MLNN, *n* (%)			0.196			0.418
No	166 (72.17)	63 (64.29)		70 (68.63)	23 (58.97)	
Yes	64 (27.83)	35 (35.71)		32 (31.37)	16 (41.03)	

TMD, tumor maximum diameter; MLNSD, maximum lymph node short diameter; MLNF, maximum lymph node fusion; MLNEV, maximum lymph node extracapsular violation; and MLNN, maximum lymph node necrosis.

**Table A2:** Performance of the clinicoradiological signature (Clinic) in five-fold cross-validation on the training cohort.

Model	Accuracy (95% CI)	AUC (95% CI)	Sensitivity (95% CI)	Specificity (95% CI)	PPV	NPV
LR	0.704 (0.655–0.754)	0.707 (0.645–0.768)	0.633 (0.580–0.685)	0.735 (0.687–0.783)	0.504	0.824
SVM	0.671 (0.620–0.722)	0.706 (0.645–0.768)	0.714 (0.665–0.763)	0.652 (0.601–0.704)	0.467	0.843
Random Forest	0.701 (0.652–0.751)	0.760 (0.702–0.819)	0.796 (0.752–0.840)	0.661 (0.610–0.712)	0.500	0.884
Extra Trees	0.637 (0.585–0.689)	0.680 (0.616–0.743)	0.663 (0.612–0.714)	0.626 (0.574–0.678)	0.430	0.814
XGBoost	0.750 (0.703–0.797)	0.775 (0.721–0.829)	0.663 (0.612–0.714)	0.787 (0.743–0.831)	0.570	0.846
LightGBM	0.729 (0.681–0.777)	0.738 (0.680–0.800)	0.643 (0.591–0.695)	0.765 (0.719–0.811)	0.538	0.834
MLP	0.613 (0.560–0.666)	0.691 (0.630–0.752)	0.755 (0.709–0.802)	0.552 (0.732–0.822)	0.418	0.841

**Table A3:** Performance of individual 2.5D deep learning (DL) models and the multiple instance learning-based signature (MIL_Rad) in five-fold cross-validation on the training set.

Model	Accuracy (95% CI)	AUC (95% CI)	Sensitivity (95% CI)	Specificity (95% CI)	PPV	NPV
**Individual 2.5D** **DL** **model**						
ResNet50	0.717 (0.839–0.911)	0.669 (0.639–0.698)	0.302 (0.261–0.344)	0.890 (0.861–0.918)	0.533	0.754
DenseNet201	0.677 (0.635–0.720)	0.628 (0.598–0.658)	0.259 (0.219–0.298)	0.852 (0.819–0.884)	0.420	0.734
VGG19	0.742 (0.703–0.782)	0.717 (0.690–0.745)	0.233 (0.194–0.271)	0.955 (0.936–0.974)	0.682	0.749
**MIL_Rad**						
LR	0.640 (0.588–0.692)	0.745 (0.689–0.801)	0.786 (0.741–0.830)	0.578 (0.525–0.632)	0.443	0.864
SVM	0.808 (0.765–0.851)	0.827 (0.775–0.880)	0.857 (0.819–0.895)	0.790 (0.743–0.831)	0.632	0.928
Random Forest	0.716 (0.668–0.765)	0.820 (0.774–0.867)	0.786 (0.741–0.830)	0.687 (0.637–0.737)	0.517	0.883
Extra Trees	0.777 (0.732–0.822)	0.846 (0.802–0.889)	0.724 (0.676–0.773)	0.800 (0.757–0.843)	0.607	0.872
XGBoost	0.823 (0.782–0.864)	0.890 (0.854–0.926)	0.776 (0.730–0.821)	0.843 (0.804–0.883)	0.679	0.898
LightGBM	0.659 (0.607–0.710)	0.788 (0.738–0.838)	0.867 (0.831–0.904)	0.575 (0.516–0.623)	0.462	0.910
MLP	0.744 (0.697–0.791)	0.784 (0.733–0.836)	0.724 (0.749–0.837)	0.752 (0.705–0.799)	0.555	0.865
