# Correction: Peng et al. CT-Based Habitat Radiomics Combining Multi-Instance Learning for Early Prediction of Post-Neoadjuvant Lymph Node Metastasis in Esophageal Squamous Cell Carcinoma. *Bioengineering* 2025, *12*, 813

**DOI:** 10.3390/bioengineering13070757

**Published:** 2026-06-29

**Authors:** Qinghe Peng, Shumin Zhou, Runzhe Chen, Jinghui Pan, Xin Yang, Jinlong Du, Hongdong Liu, Hao Jiang, Xiaoyan Huang, Haojiang Li, Li Chen

**Affiliations:** 1 Guangdong Esophageal Cancer Institute, Sun Yat-sen University Cancer Center, Guangzhou 510060, China; pengqh@sysucc.org.cn (Q.P.); yangx@sysucc.org.cn (X.Y.); liuhd@sysucc.org.cn (H.L.); 2 Department of Radiation Oncology, State Key Laboratory of Oncology in South China, Collaborative Innovation Center for Cancer Medicine, Sun Yat-sen University Cancer Center, Guangzhou 510060, China; runzhe.chen@outlook.com (R.C.); dujl@sysucc.org.cn (J.D.); huangxy@sysucc.org.cn (X.H.); 3 Department of Radiology, State Key Laboratory of Oncology in South China, Collaborative Innovation Center for Cancer Medicine, Sun Yat-sen University Cancer Center, Guangzhou 510060, China; zhousm@sysucc.org.cn; 4 Department of Radiation Oncology, Renmin Hospital, Wuhan University, Wuhan 430060, China; panjinghui@whu.edu.cn; 5 School of Electronic Information, Wuhan University, Wuhan 430064, China; jh@whu.edu.cn

## Text Correction

There was an error in the original publication [1]. In Section “3. Results”, Subsection “3.1. Clinicoradiological Signature”, an incorrect phrase was inadvertently retained during manuscript editing with tracked changes. Specifically, the phrase “with AUCs of 0.738 (95% CI: 0.678–0.797)” should have been deleted. In addition, the phrase “and was therefore selected for the subsequent analyses” has been removed to avoid potential misunderstanding. The original text “The LightGBM-based model demonstrated superior performance with AUCs of 0.738 (95% CI: 0.678–0.797) in the independent test cohort with an AUC of 0.716 (95% CI: 0.587–0.845) (Table 2) and was therefore selected for the subsequent analyses” has been corrected to “The LightGBM-based model demonstrated superior performance in the independent test cohort with an AUC of 0.716 (95% CI: 0.587–0.845) (Table 2)”.

There was also an unclear description in the original publication [1] regarding the construction of the fusion nomogram. To more clearly describe the components and construction process of the nomogram, the paragraph has been revised as follows: “The best-performing models in the independent test cohort was selected from the Habitat-Based radiomic signature and the MIL_Rad signature and subsequently integrated with independent clinicoradiological predictors using logistic regression to construct a late-fusion model. A predictive nomogram was then developed by incorporating clinicoradiological factors, the Habitat_Rad signature, and the MIL_Rad signature to predict LNM in ESCC patients undergoing neoadjuvant therapy. The nomogram, which facilitates clinical utilization, is illustrated in Figure 5. A detailed description of the construction and application of the nomogram can be found in Appendix A.5”.

In addition, the description of the models included in the performance comparison was not sufficiently clear in the original publication [1]. The sentence “The best-performing model in the test cohort from each signature, together with the late-fusion nomogram model, was included for performance comparison” has been added at the beginning of 3. Results, 3.5. Performance Comparison Among Various Signatures, Paragraph 1, to clarify that the best-performing model from each signature in the test cohort, together with the late-fusion nomogram model, was included in the comparison.

The authors state that the scientific conclusions are unaffected. This correction was approved by the Academic Editor. The original publication has also been updated.
